# *Helicobacter pylori*‐related risk predictors of gastric cancer: The latest models, challenges, and future prospects

**DOI:** 10.1002/cam4.3068

**Published:** 2020-05-04

**Authors:** Seyedeh Zahra Bakhti, Saeid Latifi‐Navid, Reza Safaralizadeh

**Affiliations:** ^1^ Department of Biology Faculty of Sciences University of Mohaghegh Ardabili Ardabil Iran; ^2^ Department of Animal Biology Faculty of Natural Sciences University of Tabriz Tabriz Iran

**Keywords:** ancestry, co‐evolution, gastric cancer, *Helicobacter pylori*, risk predictor, virulence genes

## Abstract

*Helicobacter pylori* is known as an important determinant of preneoplastic lesions or gastric cancer (GC) risk. The bacterial genotypes may determine the clinical outcomes. However, the evidence for these associations has varied between and within continents, and the actual effect of each gene and corresponding allelic variants are still debatable. In recent years, two new models have been proposed to predict the risk of GC; the phylogeographic origin of *H. pylori* strains and a disrupted co‐evolution between *H. pylori* and its human host, which potentially explain the geographic differences in the risk of *H. pylori*‐related cancer. However, these models and earlier ones based on putative virulence factors of the bacterium may not fully justify differences in the incidence of GC, reflecting that new theories should be developed and examined. Notably, the new findings also support the role of ancestry‐specific germline alteration in contributing to the ethnic/population differences in cancer risk. Moreover the high and low incidence areas of GC have shown differences in transmission ecology, largely affecting the composition of *H. pylori* populations. As a new hypothesis, it is proposed that any high‐risk population may have its own specific risk loci (or variants) as well as new *H. pylori* strains with national/maybe regional gene pools that should be considered. The latter is seen in the Americas where the rapid evolution of distinct *H. pylori* subpopulations has been occurred. It is therefore proposed that the deep sequencing of both *H. pylori* and its human host is simultaneously performed in GC patients and age‐sex‐matched controls from high‐risk areas. The expression and functional activities of the identified new determinants of GC must then be assessed and matched with human and pathogen ancestry, because some of risk loci are ancestry‐specific. In addition, potential study‐level covariates and moderator variables (eg physical conditions, life styles, gastric microbiome, etc) linked to causal relationships, and their impact, should be recognized and controlled.

## Graphical abstract

The association of *H. pylori* genotypes with the GC risk has been largely influenced by geographic and/or ethnic origin worldwide. In this regard, new models have been recently proposed to predict the risk of GC, which comprise: (a) phylogeographic origin of *H. pylori* strains and (b) disrupted co‐evolution between *H. pylori* and its human host. These models and earlier ones based on putative virulence factors of the bacterium may not fully justify differences in the incidence of GC, reflecting that new theories should be developed and examined.

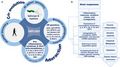

## 1 INTRODUCTION

*Helicobacter pylori* (HP) is one of the determining factors in gastric cancer (GC) incidence.^1^, ^2^ GC is one of the most common types of infection‐related cancers, as 5.5% of all cancers and more than 60% of GC cases are caused by HP infection.^3^ GC ranks the fifth among most common cancer types (5.7%) in the world and the third among cancer‐related mortality (8.2%), accounting for more than 700 000 deaths each year.^4^

The association of HP genotypes with GC risk has been widely influenced by the ethnic/geographic origin worldwide. Epidemiological studies have clarified that the prevalence of severe gastroduodenal diseases depends on HP ancestry and pathogenic factors, ethnic and geographic origin of infected people, co‐evolution between HP and its human host, host susceptibility, and environmental factors.^5^, ^6^, ^7^, ^8^, ^9^, ^10^, ^11^ In this review article, the latest models, challenges, and future directions regarding the HP‐related risk predictors of GC are discussed.

## 2 THE ASSOCIATION OF HP PREVALENCE AND ITS ANCESTRAL ORIGIN WITH THE INCIDENCE OF GC

### 2.1 The prevalence of HP infection and geographic differences in GC incidence

HP infection can be found worldwide as it has infected more than 50% of the world's population; however, the severity of HP and its related diseases significantly varies in different areas.^12^ It is shown that generally the high prevalence of HP associates with a high incidence of GC. This relation, however, has not been regularly seen in all areas of the world,in some regions, the rate of GC is low while the prevalence of HP infection is high.^13^ Despite the fact that the prevalence of HP infection is high in Africa (almost 100% of African population carries HP strains) and South Asia, the incidence of GC in these regions is very low compared to other areas of the world which is called African enigmas and Asian enigmas.^13^, ^14^ The studies have shown that the genetic properties of HP are extremely diverse concerning the ethnic‐geographic characteristics of the human hosts,^15^, ^16^ for example, HP from East Asia is considerably different from HP from Europe. The genetic diversity of HP population decreases by getting far away from Africa.^17^, ^18^, ^19^

### 2.2 Ancestral origin of HP strains and geographic differences in GC incidence

**Figure 1:**
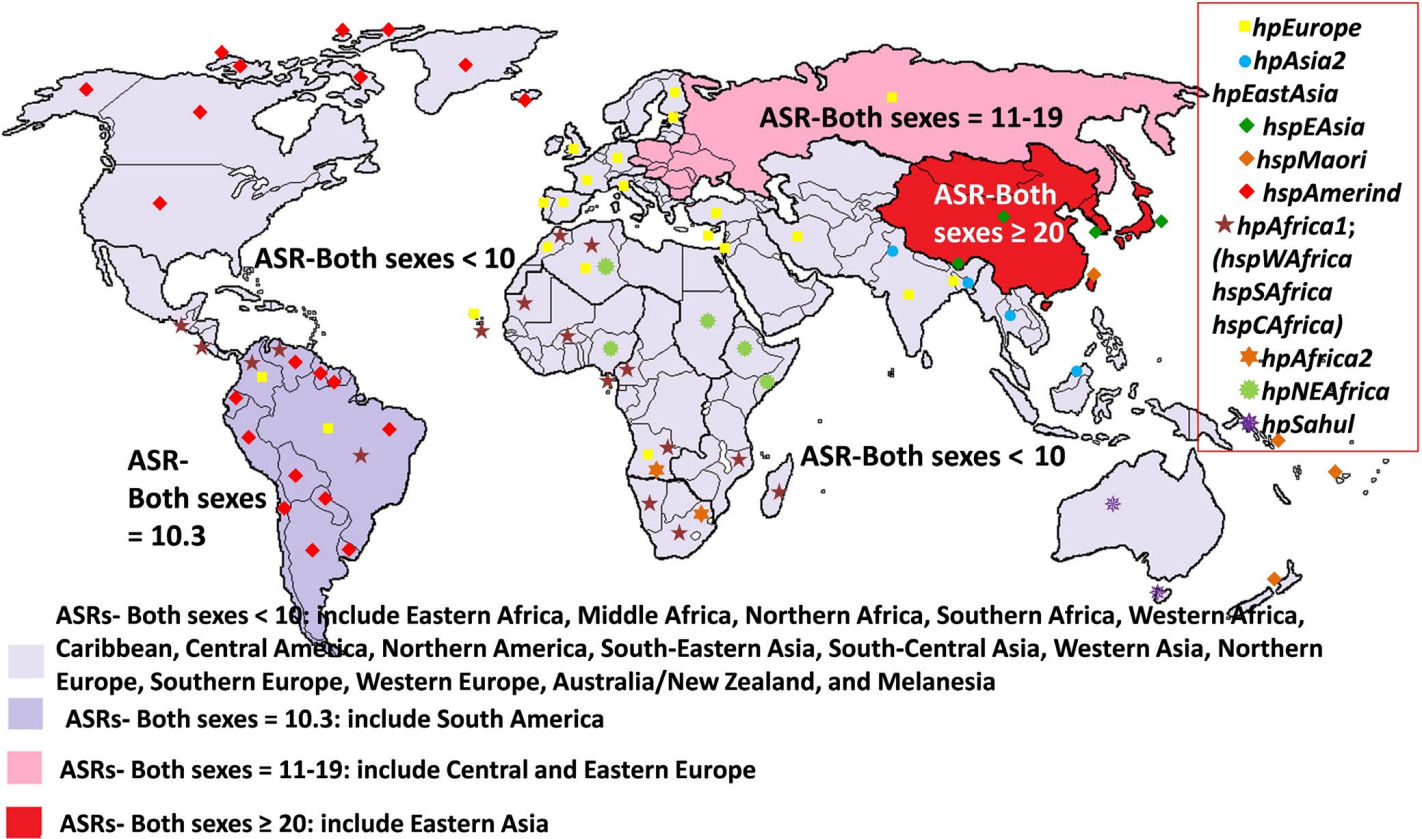
*Helicobacter pylori* ancestry and global age‐standardized incidence rates (ASR) of gastric cancer (GC). The figure explains how different *H. pylori* ancestries are distributed in the high‐ moderate‐, and low‐incidence areas of GC, and whether a particular sub‐population has a greater tendency to the areas with the high incidence of cancer. Among the bacterial ancestries, *hspEAsia* (a sub‐population of *hpEastAsia*) has been globally distributed in regions (East Asian countries) with the high incidence of GC, and *hpEurope* in areas with the low and moderate incidence of GC. The rest of populations are globally distributed in areas with low incidence of GC, which also include *hpAfrica1 (hspSAfrica, hspWAfrica, and hspCAfrica), hpAfrica2, hpNEAfrica, hpAsia2, hpSahul,* and other sub‐populations of *hpEastAsia; hspAmerind,* and *hspMaori*

Interestingly, the incidence of GC has a close association with the distribution of HP populations.^5^ The risk of cancer due to HP infection varies in the different regions of the world which can be partly attributed to the different HP genotypes circulating in various geographic regions.^20^ It has also been shown that the geographic differences in the risk of GC deriving from HP infection could be explained by the phylogeographic origin of HP strains.^5^ To date, seven bacterial population types have been observed,*hpAfrica1* (*hspSAfrica, hspWAfrica,* and *hspCAfrica*)*, hpAfrica2, hpNEAfrica, hpEurope, hpSahul, hpAsia2,* and *hpEastAsia* (*hspEAsia, hspAmerind, and hspMaori*) Table 1.^15^, ^17^, ^21^ These populations are the result of a bacterial genetic separation that led to a reduction in the recombination between strains in some populations.^13^ In Africa, most strains are *hpAfrica1, hpAfrica2 or hpNEAfrica* and in South Asia, the HP strains are mostly *hpAsia2* with a low rate of GC,while, in those areas with *hpEastAsia*, especially *hspEAsia*, the incidence of GC is so high Figure 1. Thus, East Asian strains are more related to the incidence of GC compared to other isolates designated by phylogeographic origins.^22^ European strains are highly related to the increased premalignant histologic lesions (mean 3.9, 95% CI,3.6‐4.2 for the *hpEurope* strains vs mean 2.9, 95% CI,2.5‐3.3 for the *hpAfrica1* strains, *P* = .001) and the DNA damage of human gastric epithelial cells (mean 46.0%, 95% CI, 40.1%‐51.9% for the *hpEurope* strains vs mean 22.7%, 95% CI, 13.4%‐31.9% for the *hpAfrica1* strains, *P* = .001).^5^ In Colombia, it has been shown that the population residing in the Andean highlands of Colombia who had just European ancestry were 25‐times more exposed to GC risk than the people living in the coastal region who had European and African phylogeographic origin.^5^

**Table 1:** Ancestry of global *Helicobacter pylori* strains

*H. pylori* population	Geographic distribution
*hpEurope*	Europe, Middle East, India, Bangladesh, Iran, Colombia, Portugal, Brazil, Cape Verde, Angola, Morocco, Algeria, Israel, Lebanon, Spain, France, Germany, Estonia, Finland, Russia, UK, Turkey, Palestine, Italy
*hpEastAsia*	
*hspEAsia*	East Asians, China, Japan, Korea, Bhutan
*hspMaori*	Taiwan Aboriginals, Melanesians, Polynesians
*hspAmerind*	Native Americans, North and South America, Bhutan
*hpAfrica1*	
*hspWAfrica*	Western Africa, Senegal, Colombia, Brazil, South America, Burkina Faso, Angola, South Africa, Cameroon, Morocco, Cape Verde, Costa Rica, Venezuela, Guatemala
*hspSAfrica*	South Africa, Madagascar, Brazil, Angola, South Africa, Namibia, Cameroon, Mozambique
*hspCAfrica*	Cameroon
*hpAfrica2*	South Africa, Angola
*hpNEAfrica*	Ethiopia, Somalia, Sudan, Northern Nigeria, Algeria
*hpAsia2*	Northern India, Bangladesh, Thailand, Malaysia
*hpSahul*	Australia Aboriginals and Papua New Guineans, Tasmania

In Iran, the ethnic‐geographic diversity is so high and almost 69% of Iranian people are infected with HP.^23^ In addition, it has been shown that the frequency of GC is strongly impressed by the ethnic/geographic origin.^16^ By studying the sequence of house‐keeping genes of 68 strains belonging to 9 global HP populations and 14 Iranian strains, Latifi‐Navid et al. showed that Iranian strains are in the same group as *hpEurope* population. *HpEurope* consisted of mixing the two populations of *Ancestral Europe1* (*AE1*) and *Ancestral Europe2* (*AE2*), the rates of which vary based on geographic situation. The Iranian strains have relatively equal contributions from the two ancestral sources, thus, it does not seem that there had been any sources from Iran before the expansion of populations throughout Western Eurasia.^16^ Although all the Iranian HP strains showed *hpEurope* ancestry, there were considerable differences in age‐standardized GC incidence rate within the country.^24^, ^25^

## 3 THE ASSOCIATION BETWEEN HP VIRULENCE FACTORS AND GC RISK

### 3.1 Mechanisms of carcinogenicity of HP risk factors

It seems that the prevalence of GC depends on the pathogenic factors of the bacterial strains, environmental co‐factors, and host susceptibility. Several studies have shown the considerable differences in the prevalence of the *vacA* alleles among patients with different clinical outcomes.^26^, ^27^, ^28^, ^29^ Therefore, the particular genotypes of HP may be related to the severity of gastrointestinal diseases.^9^, ^30^ Various HP pathogenic factors including *vacA*, *cagA*, *babA, hopQ, oipA*, and *homA/B* are the predictors of gastric atrophy, intestinal metaplasia, and severe clinical consequences.^30^, ^31^, ^32^, ^33^, ^34^ The *cagA* (cytotoxin‐associated gene A) and *vacA* (vacuolating cytotoxin A) genes are the two main determinants of HP‐associated disease risk that are mainly involved in the chronic gastritis and damage of epithelial cells leading to GC.^26^, ^35^ These HP CagA and VacA oncogenic proteins induce GC with a multi‐step process.^36^, ^37^ Injecting CagA into the gastric cells leads to the upregulation of oncogenes and silencing of tumor suppressor genes. It increases the level of reactive nitrogen and oxygen species, causing the DNA damage and break‐down of the cells as well as changing the microRNA expression profile. VacA results in the EGFR (epidermal growth factor receptor) expression and promotes the GC formation.^38^

### 3.2 Role of the *vacA* genotypes in the incidence of GC

One of the first pathogenic factors discovered in HP is the VacA protein that plays an important role in the bacterium establishment in the gastric mucosa and the incidence of HP‐related diseases.^39^ Although all HP strains carry the *vacA* gene, there are significant differences in the vacuole‐creating activities between strains. These differences are related to the *vacA* gene structure in the signal (s), intermediate (i), and middle (m) regions. These regions include the s1 (s1a, s1b, and s1c), s2, i1, i2, m1, and m2 subtypes.^40^ Region i plays an important role in the vacuole‐creating activity and has a strong association with GC.^9^, ^41^ This relationship is independent of and bigger than the associations of the s‐ or m‐types of *vacA* and *cagA* status with GC.^42^ Recently, one deletion of 81 bp has been found between the m and i regions and called the d region (d1 or d2). The *vacA* d1 (no deletion) genotype showed a strong association with the neutrophil infiltration and gastric mucosal atrophy in both the corpus and the antrum.^8^ The highest frequencies of the pathogenic *vacA*‐s1/‐i1 and *cagA^+^*genotypes are found in Eastern Asia (ASR—Both Sexes— = 24.2) and South‐Eastern Asia (ASR—Both Sexes— = 6), although these two regions are very different in the incidence rate of GC. The *vacA* d1 genotype is also the most abundant bacterial genotype in Eastern Asia and has not been studied in South‐Eastern Asia Table 2. However, the highest frequencies of the pathogenic *vacA* alleles (except m1) and the *cagA^+^*genotype are found in areas with the high incidence of GC (ASRs—Both Sexes—≥ 20), although there is often no association between these genotypes and the risk of GC Table 3.

**Table 2:** Prevalence (percentage) of the *H. pylori vacA* alleles and *cagA* genotype in the world

ASR‐Both Sexes (GLOBOCAN 2012)	Population of World	AM^a^ s1 (vs s2)	GM^b^, ^a^ s1 (vs s2)	AM m1 (vs m2)	GM m1 (vs m2)	AM i1 (vs i2)	GM i1 (vs i2)	AM d1 (vs d2)	GM d1 (vs d2)	AM *cagA*	GM *cagA*
6.7	South‐Central Asia	80.36%	78.93%	47.90%	41.92%	49.55%	50.06%	47.42%	49.56%	70.81%	68.62%
9.5	Western Asia	76.17%	76.02%	29.65%	29.19%	—	—	—	—	59.71%	58.83%
6.0	South‐Eastern Asia	98.43%	98.21%	60.83%	60.09%	94.52%	93.89%	—	—	89.08%	86.14%
3.4	Northern Africa	56.23%	58.03%	49.13%	32.51%	47.53%	57.62%	—	—	59.40%	51.53%
5.4	Northern Europe	93.86%	92.13%	51.44%	49.89%	—	—	—	—	59.25%	59.60%
6.3	Western Europe	74.70%	76.28%	42.00%	41.49%	—	—	—	—	71.38%	71.41%
8.6	Southern Europe	55.99%	52.32%	35.67%	31.67%	50.64%	51.25%	—	—	52.74%	49.98%
9.3	Central America	84.41%	84.28%	73.38%	76.29%	—	—	—	—	68.28%	73.56%
10.3	South America	78.03%	75.86%	73.36%	65.023%	73.33%	74.36%	—	—	72.82%	67.58%
13.5	Central and Eastern Europe	77.81%	76.78%	41.59%	40.68%	63.76%	63.86%	—	—	73.59%	63.41%
24.2	Eastern Asia	97.23%	97.76%	55.51%	49.95%	90.14%	92.55%	97.95%	97.96%	92.44%	93.23%

^a^ Arithmetic mean.

^b^ Geometric mean.

**Table 3:** Prevalence (percentage) of the *H. pylori vacA* alleles and *cagA* genotype in the low‐, moderate‐, and high‐incidence areas of gastric cancer

ASR‐both sexes (GLOBOCAN 2012)	AM^b^, ^a^ s1 (vs s2)	GM^b^ s1 (vs s2)	AM m1 (vs m2)	GM m1 (vs m2)	AM i1 (vs i2)	GM i1 (vs i2)	AM d1 (vs d2)	GM d1 (vs d2)	AM *cagA*	GM *cagA*
ASRs‐ Both sexes < 10^c^	80.32%	79.38%	49.79%	44.24%	58.66%	57.72%	53.33%	51.53%	70.31%	68.04%
ASRs‐ Both sexes = 10.3^d^	78.03%	75.86%	73.36%	65.02%	73.33%	74.36%	—	—	72.82%	67.58%
ASRs‐ Both sexes = 11‐19^e^	77.81%	76.78%	41.59%	40.68%	63.76%	63.86%	—	—	73.59%	63.41%
ASRs‐ Both sexes ≥ 20^f^	97.23%	97.76%	55.51%	49.95%	90.14%	92.55%	97.95%	97.96%	92.44%	93.23%

^a^ Arithmetic mean.

^b^ Geometric mean.

^c^ Include Eastern Africa, Middle Africa, Northern Africa, Southern Africa, Western Africa, Caribbean, Central America, Northern America, South‐Eastern Asia, South‐Central Asia, Western Asia, Northern Europe, Southern Europe, Western Europe, Australia/New Zealand, and Melanesia.

^d^ Include South America.

^e^ Include Central and Eastern Europe.

^f^ Include Eastern Asia.

Heterogeneity in the *vacA* gene might be an important risk factor in the creation of different gastrointestinal diseases, especially GC, in HP*‐*infected patients.^9^, ^26^, ^43^, ^44^, ^45^ HP strains with the *vacA* s1, i1, m1, or d1 genotypes from Western countries were strongly associated with the risk of GC (adjusted ORs: 3.17, 8.57, 10.65, and 8.04, respectively). In Spanish patients, infection with the HP strains representing the *vacA* s1m1*/cagA^+^*genotype has strongly increased the progression of gastric precancerous lesions compared with the strains carrying the *vacA* s2m2*/cagA* ^−^ genotype (OR = 4.80).^6^ Moreover, it is proposed that the d region genotype (d1 vs d2) is an important risk locus for GC in Western strains.^8^ In contrast, in the HP strains from Eastern countries, there was no significant association between the *vacA* polymorphisms, clinical consequences, and histopathologic patterns.^8^

A new polymorphism region at the 3 ʹ‐end of the *vacA* gene has been recently identified and named as the *vacA* c (c1: with deletion of 15 bp) region. The *vacA* c1‐type was significantly associated with the risk of GC. This association was independent of and larger than the associations of the i1‐, m1‐, and d1‐types of the *vacA* alleles or *cagA* status with GC.^26^ We also reported that the *vacA* c1 genotype had an intense association with the risk of cardia gastric adenocarcinoma (CGA), non‐CGA, diffuse‐type gastric adenocarcinoma (DGA), and intestinal‐type gastric adenocarcinoma (IGA), whether the controls were non‐tumor (ie those with non‐atrophic gastritis (NAG) and PUs,the OR was 14.11, 9.59, 11.91, and 16.93, respectively) or those with NAG (the OR was 10.71, 8.11, 9.56, and 11.22, respectively).^46^

Little is known about all the constellations of five polymorphic sites of *H. pylori vacA* and *cagA* status. Recently, we found novel five‐ and six‐genotype combinations (*vacA* s1m1i1d1c1, s1m2i1d2c1, s1m2i2d2c1, and s1m2i2d2c1*cagA*) associated with the risk of GC. The ORs were 2.433, 11.524, 4.200, and 6.263, respectively. These associations were largely dependent on the presence of c1‐type of *vacA*. Therefore, analysis of all the combined genotypes of the *vacA* alleles and the *cagA* status may play an important role in determining clinical outcomes associated with *H. pylori*.^47^

The detailed information regarding the associations of HP *vacA* gene polymorphisms with the risk of GC in low‐, moderate‐, and high‐incidence areas are indicated in Table 4. In most areas, the association of some HP *vacA* alleles with the risk of GC has been demonstrated. However, most studies in East Asia with the high incidence of GC did not show such a significant association, due mainly to the high prevalence of the pathogenic *vacA* alleles in this area.

**Table 4:** Associations of the *H. pylori vacA* alleles and *cagA* genotype with the risk of gastric cancer in the low‐, moderate‐, and high‐incidence areas of gastric cancer

ASR‐Both sexes (GLOBOCAN 2012)	Population of World	Country	Study/ Publication Time	Genotype(s) Related to GC^a^	*P* = value	OR (95% CI)
6.7	South‐Central Asia	Iran/ Ardabil	Abdi et al. (2017)^102^	*vacA* i1	.046	3.00 (1.08‐8.32)
				*vacA* d1	.020	3.25 (1.21‐8.74)
				*vacA* m1d1	.038	4.28 (1.24‐14.73)
				*vacA* m1i1d1	.028	4.82 (1.21‐19.21)
		Iran	Bakhti et al. (2016)^26^	*vacA* m1	1.34e‐04	4.29 (2.03‐9.08)
				*vacA* i1	2.52e‐05	6.11 (2.63‐14.19)
				*vacA* d1	.003	3.18 (1.49‐6.76)
				*vacA* c1	1.89e‐08	15.13 (5.86‐39.01)
				*cagA*+	.03	2.59 (1.09‐6.12)
				*vacA* i1c1	1.47e‐06	43.44 (9.35‐201.73)
				*vacA* m1i1c1	4.00e‐06	37.77 (8.07‐176.85)
		Iran	Rhead et al. (2007)^9^	*vacA* s1	<.05	—
				*vacA* m1	<.05	—
				*vacA* i1	<.0005	—
				*cagA*+	<.005	—
		Iran	Basiri et al. (2014)^78^	*vacA* d1	.015	4.662 (1.345‐16.164)
		Iran	Mottaghi et al. (2014)^79^	*vacA* i1	.0	13.142 (3.116‐55.430)
		Afghanistan	Yakoob et al. (2013)^103^	*vacA* s1a/b1	.033	—
				*cagA*+	.006	—
				*vacA* s1b/m1	.007	—
				*cagA*+/*vacA* s1a/m1	.006	—
		Pakistan	Yakoob et al. (2013)^103^	*vacA* s1a	.006	—
				*vacA* s1a/m1	.002	—
				*cagA*+/*vacA* s1a/m1	.001	—
		Pakistan	Khan et al. (2013)^104^	*cagA*+	<.0005	—
				*vacA* s1am1	<.05	—
9.5	Western Asia	Turkey	Saribasak et al. (2004)^105^	*cagA*+	<.001	—
		Kurdistan Region, Northern Iraq	Rasheed^106^	*vacA* d1	.002	—
3.4	Northern Africa	morocco	El Khadir et al. (2017)^107^	*vacA* m1	<.001	—
				*vacA* i1	<.001	—
				*vacA* i1m1	<.001	29.73 (5.08‐173.73)
6.3	Western Europe	Belgium	Memon et al. (2014)^108^	*vacA* s1	.01	9.37 (1.16 −201.89)
				*vacA* i1	.003	12.08 (1.50‐259.64)
				*cagA*+	<.05	Infinity (0.76‐infinity)
				*vacA* s1/i1	<.02	—
		Germany	Miehlke et al. (2000)^109^	*vacA* m1	.005	—
				*cagA*+	.01	—
				*vacA* s1m1	.005	—
8.6	Southern Europe	Portugal	Ferreira et al. (2012)^110^	*vacA* i1	<.001	22 (7.9‐63)
		Italy	Basso et al. (2008)^43^	*vacA* s1	<.001	8.28 (2.75‐24.95)
				*vacA* m1	<.05	5.25 (1.03‐25.80)
				*vacA* i1	<.001	5.02 (2.10‐11.98)
				*cagA*+	<.05	11.80 (3.43‐40.61)
9.3	Central America	Southern Mexico	Roman‐Roman et al. (2017)^111^	*vacA* s1m1	.001	6.58 (2.15‐20.08)
10.3	South America	northern region of Brazil	Vinagre RMDF et al. (2018)^112^	*vacA* s1m1/*cagA*+	.001	G = 62.52
13.5	Central and Eastern Europe	Russian/ Vladivostok	Stenkova et al. (2013)^113^	*vacA* s1	<.04	1.3‐fold
				*vacA* s1m1	—	—
24.2	Eastern Asia	China	Wei et al. (2012)^114^	*vacA* s1cm2	<.05	—
		Okinawa, Japan	Matsunari et al. (2012)^82^	*vacA* s1	.03	—
				*vacA* m1	.01	—
				*cagA*+	.04	—
				East‐Asian‐type *cagA*	.01	—
				*vacA* s1m1	.006	—
				East‐Asian‐type *cagA*/*vacA* s1m1	.003	—

^a^ For each study, all the single genotypes associated with GC were considered. About the two‐ and three‐genotype combinations, the ones with the highest OR values were expressed.

### 3.3 Role of the *cagA* genotype in the incidence of GC

*cagA* is another pathogenic gene of HP^48^ and *cagA* ^+^ strains are more pathogenic than *cagA* ^−^ strains.^49^ In this regard, epidemiological studies have disclosed that the attendance of the *cagA* is associated with the development of gastric precancerous lesions, GC,^50^, ^51^ increased mucosal inflammation,^52^ and cell proliferation,^53^ however, other reports have not shown such a relation.^54^, ^55^ It is reported that more than 90%‐100% of HP strains from East Asian countries are *cagA^+^*.^56^, ^57^ However, this high prevalence of the *cagA* ^+^ genotype is independent of its association with clinical consequences. In contrast, just 50%‐70% of HP strains from Western countries have the *cagA* ^+^ genotype.^43^, ^56^, ^58^, ^59^ Given the reports from Western countries, it is likely that the *cagA^+^* genotype may be a useful biomarker for prediction of clinical outcomes,however, in Eastern countries, this issue requires more investigations. It seems that the determination of the number of phosphorylation sites of CagA is more important than the detection of the *cagA* genotype alone.^59^, ^60^ A large epidemiological study of 2145 patients in Venezuela (in Latin America,a region with the high incidence of GC and HP infection) also showed a strong association between the *cagA*‐positive genotype and the severity of gastric precancerous lesions. The odds ratios were increased with the severity of the disease. The OR was 2.00 for chronic gastritis, 7.35 for intestinal metaplasia type II and 16.7 for dysplasia.^61^ Moreover, a large cross‐sectional study in China (Linqu County) showed that individuals seropositive to Omp, CagA, VacA, and HP0305 significantly increased the risk of precancerous lesions (ORs = 5.37, 3.23, 3.75, and 3.85, respectively). The risk estimate was increased more than fourfold by seropositivity to 4, 5, or more than 6 specific HP antigens (Omp, HP0305, HyuA, HpaA, CagA, and VacA) relative to seropositivity to 3 or less than 3 antigens. However, the risk was increased to 7.43 when the individuals were seropositive to both Omp and HP0305. This indicate the fact that in East Asia these serological biomarkers are more important than CagA seropositivity alone for predicting the risk of advanced gastric lesions.^62^ The detailed information regarding the association of the HP *cagA^+^* genotype with the risk of GC in low‐, moderate‐, and high‐incidence areas are indicated in Table 4.

### 3.4 Wide diversity at the EPIYA and CM motifs of CagA and the risk of GC

CagA protein from various HP strains has a wide diversity at the carboxyl terminus that includes repetitive phosphorylation sites (EPIYA motif).^59^ The reports have shown that these repetitive EPIYA sites play a major role in the development of gastrointestinal disorders.^59^, ^63^ The diversity of EPIYA motif can influence the CagA structure and its interaction inside the gastro‐epithelial cells.^64^ Based on the sequences surrounding the EPIYA motif, EPIYA‐A, EPIYA‐B, EPIYA‐C, or EPIYA‐D segments have been specified.^65^, ^66^ The number and combination of various motifs, which vary according to geographic locations, determine clinical consequences.^11^, ^43^, ^67^, ^68^ There have been associations between the different motifs of the CagA protein, the origin country of each sequence type (ST), and the prevalence of gastrointestinal disorders.^11^ The EPIYA‐ABD type of CagA (East Asian‐type) is more virulent compared to the EPIYA‐ABC type (Western‐type) and has a stronger association with the GC risk.^56^, ^69^ In Northeastern Thailand, it has been reported that most strains represent Western‐type CagA and are significantly associated with PUD.^70^ However, they had no relation with non‐ulcer dyspepsia and GC, consistent with previous studies.^51^, ^70^ Furthermore, the recent study conducted in East China showed that EPIYA‐ABD had no relation with gastric diseases (chronic gastritis and gastric or duodenal ulcerations), and only polymorphism at amino‐acids 878 and 879, flanking the EPIYA‐A motif, had a significant association with GC.^54^ It seems that the number of motifs/segments of EPIYA, especially EPIYA‐C is associated with the risk of atrophy,^71^ GC,^11^, ^43^, ^72^ and intestinal metaplasia.^43^

The CagA multimerization motif (CM)—located within the EPIYA‐C motif and downstream of the EPIYA‐D motif—is responsible for the dimerization of CagA^73^ and categorized as Eastern and Western based on CM sequences.^74^ The type and number of CM motifs may influence the multimerization of CagA.^73^ The CagA protein with two Western CM motifs (vs. that with the Western and Eastern CM motifs) causes cell elongation^74^, ^75^ and a greater affinity for SHP‐2.^74^ The Western CagA proteins with an Eastern CM motif may reduce the multimerization and CagA pathogenicity.^75^ Unlike CagA EPIYA motifs, the association of CM motifs with gastrointestinal disorders has not been well studied. However, the presence of one or two Western CM motifs in the absence of an Eastern CM motif was linked with the risk of GC.^76^

## 4 THE CONFLICTS BETWEEN THE LATEST MODELS IN PREDICTING THE RISK OF GC

### 4.1 Ancestral origin or pathogenic HP factors: Which is more important in GC?

Sablet et al showed that the ancestry of HP is associated with the incidence of GC risk. The Colombian people infected by the *hpEurope* strains showed more severe tissue damage compared to people infected by *hpAfrica1* strains.^5^ We have shown that although Iranian HP strains represent European ancestry with almost equal contributions from two ancestral sources (*AE1* and *AE2*), a difference of 2‐ to 10‐times were observed in the GC incidence between Southern and Northwestern‐Northern regions of Iran.^16^, ^77^ This difference was not consistent with the new prediction model of GC based on bacterial ancestry, thus it has still remained obscure. However, it was proposed that the *vacA* d1/‐i1 genotypes have an excellent potential for differentiating HP strains from the low‐ and high‐incidence regions of GC in Iran.^77^ A strong association was found between the HP *vacA* i1‐/ d1‐genotypes and the risk of GC in East Azerbaijan region (North‐West Iran), where the GC risk is high in males.^78^, ^79^ Yamaoka et al have also previously studied the relationship between HP pathogenic factors such as *cag*PAI, *oipA*, *babA*, *iceA,* and *vacA* from Colombian people and their clinical consequences. Although *cag*PAI and *oipA* were associated with each other, just the *oipA* gene was inclined toward association with GC.^33^ Therefore, they proposed that the *oipA* in *cagA*‐positive strains might improve the geographic origin diversity which is specified by multi‐locus sequence typing (MLST) analysis, challenging the Sablet's model.^80^

As a result, the difference in tissue damage might be due to the various status of the *oipA* gene; therefore, it is required to study the relationship between the pathogenic factors and phylogenic tree by MLST. Also, it was stated that a selection bias was possible in Sablet's model as they merely considered 64 participants who were suffered from *cagA* ^+^/*vacA* s1m1 strains and did not focus on those suffering from *cagA* ^−^ genotype. In fact, all *cagA* ^−^ strains were related to *hpEurope,* and a very low amount of tissue damage was observed in patients infected by these strains. It was stated that the *hpEurope* strains may manifest low clinical pathogenesis in the absence of *cagA*. Therefore, this factor could better predict GC risk in comparison with phylogeographic origin.^80^ Shiota et al proposed another case from Okinawa in Japan, which challenged the Sablet's model. Many Okinawa strains have been categorized into three *hpEastAsia* clusters. A main difference between clinical outcomes and phylogeographic origin has been presented,however, the clinical results were dependent on the *cagA* (*cagA* ^−^, Western‐type, and East Asian‐type) status and the *vacA* m1 or m2 genotypes; GC was prevalent in many clusters which mainly had the *cagA* of East Asian‐type. The MLST analysis revealed that when only the *cagA* of Western‐type or *cagA* ^−^ strains were applied, there was not any difference in the GC prevalence, indicating that *cagA* better predicts GC in comparison with phylogeographic origin.^80^ Nevertheless, Okinawa in Southwestern Japan may show a lower GC incidence than expected from other areas of the country. Thus, the data cannot be generalized to Japan as a whole. In addition, Shiota et al conducted a study in Andean region of Colombia showing that the MLST‐based phylogeographic origin is not enough to specify the GC risk in the *hpEurope* strains. Hence, the phylogenetic analysis of HP virulence factors is essential for diagnosing clinical findings.^81^ However, the phylogenetic tree achieved by conducting MLST did not correlate with the *cagA* or *vacA* types, though the *vacA* s2m2 or *cagA* ^−^ strains were clustered relatively, proposing that *vacA* and *cagA* genotypes cannot be assumed as indicating factors for phylogenetic origin based on MLST. They found that the *cagA* status was associated with a phylogenetic cluster.^80^, ^82^

Another study was carried out in Bangladesh which is a country in South Asia with a lower incidence of GC and a higher prevalence of HP infection (60.2%); it was shown that HP strains from Bengali population had two main *hpAsia2* and *hpEurope* populations. The study demonstrated that the *hpEurope* population was associated with a higher GC risk in comparison to the *hpAsia2* population. However, the *hpAsia2* strains were strongly associated with an inflammation in the antrum compared to the *hpEurope* populations. This may be as a result of a higher proportion of less‐pathogenic genotypes of the *vacA* and *cagA* genes in the *hpEurope* strains associated with a low histologic damage. Nevertheless, independent of the HP population types, strains with the *vacA* s1m1i1d1c1 genotype significantly caused greater inflammatory responses and atrophy in the antrum, unlike strains with the *vacA* s2m2i2d2c2, s2m2i2d2c2, or s1m2i1d1c2 genotypes. Bangladesh strains were categorized into two different HP populations with various genotypes. The low incidence of GC in Bangladesh is possibly because of a great number of less‐virulent genotypes, which probably better predict the GC risk in comparison with the ancestral origin of HP strains. Thus, the findings may justify the “*Asian enigma*” in Bangladesh, with the higher HP infection prevalence and the lower GC risk.^14^

### 4.2 Interaction and co‐evolution between HP and host ancestries can be the main determining risk factor for GC

It has been shown that in Western countries, *cagA* positivity is associated with an increased risk of GC; however, in East Asian countries, although CagA represents EPIYA‐D segment and is biologically stronger, its presence does not indicate an elevated risk of GC.^6^, ^8^, ^9^, ^35^, ^55^ In this regard, no significant relationship was found between the *vacA* genotypes, clinical consequences, and histopathologic variations in East Asian countries.^8^, ^83^ Clustering analyses have isolated six clinical Colombian strains from the regions with the higher and the lower prevalence of GC on the basis of geographic origin,the European strains showed the higher expression of the BabB, CagA, and VacA pathogenic factors and were correlated with the increased histopathologic lesions. European strains remarkably increased the expression of interleukin 8 in AGS cells compared to the African strains. The African strains considerably promoted apoptosis,while just one strain with the European origin considerably promoted apoptosis. These data proposed that the difference in the expression of virulence factors is resulted by the host‐pathogen interactions and might have a remarkable role in carcinogenesis.^10^ Increased expression of BabB, CagA, and VacA and decreased apoptosis in the European strains might indicate an increased damage to DNA and suggest GC risk in individuals infected with the European strains compared with those infected with the African strains.^5^ Thus, it is assumed that the bacterial pathogenic factors which are determined by the phylogeographic origin of the HP strains might increase the risk of GC.^10^ Bhutan, a South Asian country, has a high incidence of GC and HP infection in comparison to the neighborhood countries, like India. According to phylogeographic analyses, Indian strains are of the *hpAsia2* and/ or *hpEurope* types and have Western‐type CagA. However, most Bhutan strains are from the East Asian population (though some strains are a mixture of Amerindian and East Asian population types or relate to the European type) and mostly (90%) have East Asian‐type CagA (AB'BD and AB'BBD), all strains have *vacA* s1 type. The difference in the incidence of GC between Bhutan and India is the result of the differences in the phylogeographic origin and virulence factors of HP. HP strains of Bhutan with East Asian‐type CagA showed significantly increased inflammatory responses compared to those with Western‐type CagA. This justifies the higher GC risk in Bhutan in comparison to India.^84^

Kodaman et al studied two Colombian populations from different geographic regions with the same prevalence of HP infection, but an extremely different incidence of GC (150/100 000 vs 6/100 000). They showed that the *cagA* gene alone cannot be a reason for the difference in the risk of gastric precancerous lesions between these two populations and the interaction between bacterial and host ancestries can be the reason for this difference. The human host and HP genetics are both effective in pathogenesis; however, the impact of human ancestry on histopathology is dependent on the HP ancestry, and vice versa. In this model, African HP ancestry was comparatively benign in people of African ancestry; however, it was detrimental in people with substantial Amerindian ancestry, reflecting the fact that disease risk is modulated by co‐evolution. Thus, the disrupted co‐evolution between HP and human host could explain why the GC incidence is high in the population of Andes Mountains. When the genomic diversity of the human host and pathogen are analyzed together, it will be more predictive of histopathology compared to the time when just one of them is analyzed. Accordingly, it seems that the co‐evolutionary relationships are the main determining risk factors for gastric diseases.^7^

Recently, Thorell et al have investigated HP evolution during the recent colonization of Americas. In the United States, strains showing African and European ancestry have remained genetically distinct. In contrast, in Colombia and Nicaragua, bottlenecks and widespread genetic exchange among isolates have led to the rapid emergence of new HP subpopulations. Three outer membrane proteins showing atypical levels of Asian ancestry were found in American strains. In contrast, the alleles that were almost exclusively fixed in South American isolates were found. Thus, the ethnic composition of hosts may play an important role in the colonization of incoming strains. Moreover the high and low incidence areas of GC may show differences in transmission ecology, largely affecting the composition of HP populations.^85^

**Figure 2:**
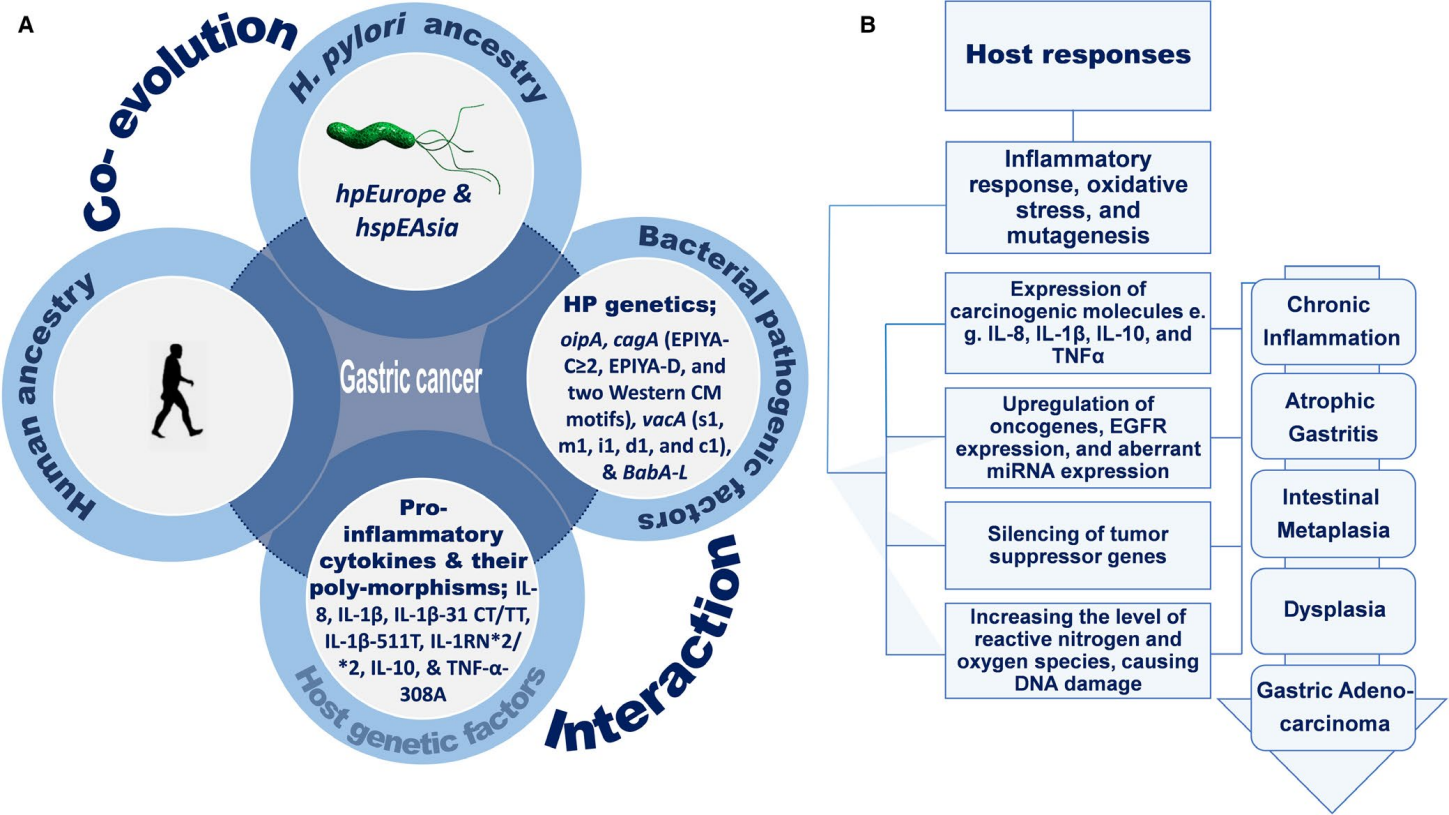
A, Interaction and co‐evolution between *H. pylori* and human host in gastric cancer (GC) susceptibility. B, Host responses and risk for GC progression

Differences in the host risk genotypes, like pro‐inflammatory cytokine polymorphisms, in alliance with the infection of HP strains were associated with the risk of GC in many populations.^86^, ^87^ Therefore, genetic variances of both HP strains and the host may demonstrate the difference in GC risk in mountainous areas.^88^ In areas with greater GC incidence, HP infection is observed with strains having virulence factors linked to increased inflammatory response, atrophic gastritis, and an elevated GC risk. Hence, HP‐host cell interactions via the induction of genetic instability of the host genome (double‐stranded breaks, mismatch repair, methylation, etc) and aberrant miRNA expression result in GC.^89^ Moreover, IL‐1β significantly amplifies inflammatory response during HP infections^90^, ^91^ and IL‐1β‐31 CT and TT may increase possibility of the infection by HP.^92^ The IL‐1β‐511T, IL‐1RN*2/*2, IL‐10 (haplotype ATA/ATA), and TNF‐α‐308A genotypes were significantly associated with an increased risk of non‐cardia GC with HP infection.^93^, ^94^ Furthermore, the *cagA*(+)/*vacA* s1(+)/IL‐1β‐511T genotype increased the risk of severe gastric anomalies.^95^ These findings, in turn, emphasize that the cytokine gene polymorphisms should be considered in GC host‐pathogen interaction models Figure 2.

## 5 FUTURE DIRECTIONS

In recent years, it has been proposed two new models to predict GC; the phylogeographic origin of HP strains and a disrupted co‐evolution between HP and its human host. As discussed above, several studies have challenged the first new model and proposed the virulence factors as the best predictors of GC.^33^, ^77^, ^80^ In addition, the strains from populations of East Asian countries such as Korea and Japan, with an extremely high incidence of GC, belonged to the *hspEAsia* group.^15^, ^17^ In these populations, both the host and bacterial ancestries have remained relatively pure,^15^, ^17^, ^96^, ^97^ reflecting the fact that the disrupted co‐evolution is unlikely to occur, challenging the Kodaman's model. Therefore, these models and earlier ones based on putative pathogenic factors of the HP may not fully justify differences in the incidence of GC.

In recent years, the genetic studies of complex phenotypes are shifting to include the lower frequencies of rare variants, which is dependent on ‘common disease–rare variant’ hypothesis and can only be allowed by an advanced sequencing technology, namely next‐generation sequencing (NGS). This postulates that the multiple rare variants which represent the larger effect sizes are the important determinants of disease heritability. In contrast, genome‐wide association studies (GWASs) are primarily examining the ‘common disease–common variant’ hypothesis, which is based on the principle of linkage disequilibrium. Therefore, exome sequencing combined with genotyping arrays is probably the best approach over the next decade to explore cancer‐risk loci.^98^, ^99^, ^100^ Notably, the new findings also support the role of ancestry‐specific germline alteration in contributing to the ethnic/population differences in cancer risk.^101^ Moreover, the high and low incidence areas of GC have shown differences in transmission ecology, largely affecting the composition of HP populations.^85^ As a new hypothesis, it is proposed that any high‐risk population may have its own specific risk loci (or variants) as well as new HP strains with national/maybe regional gene pools that should be considered. The latter is seen in the Americas where the rapid evolution of distinct HP subpopulations has been occurred.^85^ It is therefore proposed that the deep sequencing of both HP and its human host is simultaneously performed in GC patients and age‐sex‐matched controls from high‐risk areas. The expression and functional activities of the identified new determinants of GC must then be assessed and matched with human and pathogen ancestry because some risk loci are ancestry‐specific.^98^, ^101^ In addition, potential study‐level covariates and moderator variables (eg physical conditions, life styles, gastric microbiome, etc) linked to causal relationships, and their impact, should be recognized and controlled.

## CONFLICT OF INTEREST

No conflict of interest to be declared.

## AUTHOR’S CONTRIBUTIONS

SLN and RS provided direction in the preparation of the manuscript. SZB and SLN performed primary literature search. SZB and SLN wrote the first draft of manuscript. SLN, SZB, and RS discussed and revised the manuscript. SZB managed the references. SLN approved the version to be published.

## Data Availability

The data that support the findings of this study are available from the corresponding author upon reasonable request.

## References

[1] Suerbaum S , Michetti P . Helicobacter pylori infection. N Engl J Med. 2002;347:1175‐1186. 12374879 10.1056/NEJMra020542

[2] Abdi E , Latifi‐Navid S , Zahri S , Yazdanbod A , Pourfarzi F . Risk factors predisposing to cardia gastric adenocarcinoma: Insights and new perspectives. Cancer Med. 2019;8(13):6114–6126. 31448582 10.1002/cam4.2497 PMC6792520

[3] Parkin DM . The global health burden of infection‐associated cancers in the year 2002. Int J Cancer. 2006;118:3030‐3044. 16404738 10.1002/ijc.21731

[4] Bray F , Ferlay J , Soerjomataram I , Siegel RL , Torre LA , Jemal A . Global cancer statistics 2018: GLOBOCAN estimates of incidence and mortality worldwide for 36 cancers in 185 countries. CA Cancer J Clin. 2018;68:394‐424. 30207593 10.3322/caac.21492

[5] de Sablet T , Piazuelo MB , Shaffer CL , et al. Phylogeographic origin of Helicobacter pylori is a determinant of gastric cancer risk. Gut. 2011;60:1189‐1195. 21357593 10.1136/gut.2010.234468 PMC3133872

[6] Gonzalez CA , Figueiredo C , Lic CB , et al. Helicobacter pylori cagA and vacA genotypes as predictors of progression of gastric preneoplastic lesions: a long‐term follow‐up in a high‐risk area in Spain. Am J Gastroenterol. 2011;106:867‐874. 21285949 10.1038/ajg.2011.1

[7] Kodaman N , Pazos A , Schneider BG , et al. Human and Helicobacter pylori coevolution shapes the risk of gastric disease. Proc Natl Acad Sci USA. 2014;111:1455‐1460. 24474772 10.1073/pnas.1318093111 PMC3910595

[8] Ogiwara H , Sugimoto M , Ohno T , et al. Role of deletion located between the intermediate and middle regions of the Helicobacter pylori vacA gene in cases of gastroduodenal diseases. J Clin Microbiol. 2009;47:3493‐3500. 19726606 10.1128/JCM.00887-09 PMC2772588

[9] Rhead JL , Letley DP , Mohammadi M , et al. A new Helicobacter pylori vacuolating cytotoxin determinant, the intermediate region, is associated with gastric cancer. Gastroenterology. 2007;133:926‐936. 17854597 10.1053/j.gastro.2007.06.056

[10] Sheh A , Chaturvedi R , Merrell DS , Correa P , Wilson KT , Fox JG . Phylogeographic origin of Helicobacter pylori determines host‐adaptive responses upon coculture with gastric epithelial cells. Infect Immun. 2013;81:2468‐2477. 23630959 10.1128/IAI.01182-12 PMC3697613

[11] Xia Y , Yamaoka Y , Zhu Q , Matha I , Gao X . A comprehensive sequence and disease correlation analyses for the C‐terminal region of CagA protein of Helicobacter pylori. PLoS ONE. 2009;4:e7736. 19893742 10.1371/journal.pone.0007736 PMC2768901

[12] Everhart JE . Recent developments in the epidemiology of Helicobacter pylori. Gastroenterol Clin North Am. 2000;29:559‐578. 11030073 10.1016/s0889-8553(05)70130-8

[13] Dong QJ , Zhan SH , Wang LL , Xin YN , Jiang M , Xuan SY . Relatedness of Helicobacter pylori populations to gastric carcinogenesis. World J Gastroenterol. 2012;18:6571‐6576. 23236231 10.3748/wjg.v18.i45.6571 PMC3516211

[14] Aftab H , Miftahussurur M , Subsomwong P , et al. Two populations of less‐virulent Helicobacter pylori genotypes in Bangladesh. PLoS ONE. 2017;12:e0182947. 28797101 10.1371/journal.pone.0182947 PMC5552282

[15] Falush D , Wirth T , Linz B , et al. Traces of human migrations in Helicobacter pylori populations. Science. 2003;299:1582‐1585. 12624269 10.1126/science.1080857

[16] Latifi‐Navid S , Ghorashi SA , Siavoshi F , et al. Ethnic and geographic differentiation of Helicobacter pylori within Iran. PLoS ONE. 2010;5:e9645. 20339588 10.1371/journal.pone.0009645 PMC2842290

[17] Linz B , Balloux F , Moodley Y , et al. An African origin for the intimate association between humans and Helicobacter pylori. Nature. 2007;445:915‐918. 17287725 10.1038/nature05562 PMC1847463

[18] Manica A , Prugnolle F , Balloux F . Geography is a better determinant of human genetic differentiation than ethnicity. Hum Genet. 2005;118:366‐371. 16189711 10.1007/s00439-005-0039-3 PMC1805472

[19] Ramachandran S , Deshpande O , Roseman CC , Rosenberg NA , Feldman MW , Cavalli‐Sforza LL . Support from the relationship of genetic and geographic distance in human populations for a serial founder effect originating in Africa. Proc Natl Acad Sci USA. 2005;102:15942‐15947. 16243969 10.1073/pnas.0507611102 PMC1276087

[20] Yamaoka Y , Kato M , Asaka M . Geographic differences in gastric cancer incidence can be explained by differences between Helicobacter pylori strains. Intern Med. 2008;47:1077‐1083. 18552463 10.2169/internalmedicine.47.0975 PMC3732488

[21] Moodley Y , Linz B , Yamaoka Y , et al. The peopling of the Pacific from a bacterial perspective. Science. 2009;323:527‐530. 19164753 10.1126/science.1166083 PMC2827536

[22] Suzuki R , Shiota S , Yamaoka Y . Molecular epidemiology, population genetics, and pathogenic role of Helicobacter pylori. Infect Genet Evol. 2012;12:203‐213. 22197766 10.1016/j.meegid.2011.12.002 PMC3294018

[23] Nouraie M , Latifi‐Navid S , Rezvan H , et al. Childhood hygienic practice and family education status determine the prevalence of Helicobacter pylori infection in Iran. Helicobacter. 2009;14:40‐46. 19191895 10.1111/j.1523-5378.2009.00657.x

[24] Eskandar H , Hossein SS , Rahim M , Jalal H , Mehrdad A , Rajabi T . Clinical profile of gastric cancer in Khuzestan, southwest of Iran. World J Gastroenterol. 2006;12:4832‐4835. 16937464 10.3748/wjg.v12.i30.4832 PMC4087616

[25] Mohebbi M , Mahmoodi M , Wolfe R , et al. Geographical spread of gastrointestinal tract cancer incidence in the Caspian Sea region of Iran: spatial analysis of cancer registry data. BMC Cancer. 2008;8:137. 18479519 10.1186/1471-2407-8-137 PMC2397428

[26] Bakhti SZ , Latifi‐Navid S , Mohammadi S , et al. Relevance of Helicobacter pylori vacA 3'‐end region polymorphism to gastric cancer. Helicobacter. 2016;21:305‐316. 26612250 10.1111/hel.12284

[27] Gholizadetobnagh S , Bakhti SZ , Latifi Navid S , Zahri S , Sadat Bakhti F . Role of plasticity region genes and cagE gene of cagPAI of Helicobacter pylori in development of gastrointestinal (GI) diseases. Asian Pac J Cancer Prev. 2017;18:43‐49. 28240008 10.22034/APJCP.2017.18.1.43 PMC5563118

[28] Gonzalez‐Valencia G , Atherton JC , Munoz O , Dehesa M , la Garza AM , Torres J . Helicobacter pylori vacA and cagA genotypes in Mexican adults and children. J Infect Dis. 2000;182:1450‐1454. 11023467 10.1086/315864

[29] Raei N , Latifi‐Navid S , Zahri S . Helicobacter pylori cag Pathogenicity Island cagL and orf17 genotypes predict risk of peptic ulcerations but not gastric cancer in Iran. Asian Pac J Cancer Prev. 2015;16:6645‐6650. 26434889 10.7314/apjcp.2015.16.15.6645

[30] Al‐Maleki AR , Loke MF , Lui SY , et al. Helicobacter pylori outer inflammatory protein A (OipA) suppresses apoptosis of AGS gastric cells in vitro. Cell Microbiol, 2017;19 1–24. 10.1111/cmi.12771 28776327

[31] Jung SW , Sugimoto M , Graham DY , Yamaoka Y . homB status of Helicobacter pylori as a novel marker to distinguish gastric cancer from duodenal ulcer. J Clin Microbiol. 2009;47:3241‐3245. 19710266 10.1128/JCM.00293-09 PMC2756951

[32] Ohno T , Sugimoto M , Nagashima A , et al. Relationship between Helicobacter pylori hopQ genotype and clinical outcome in Asian and Western populations. J Gastroenterol Hepatol. 2009;24:462‐468. 19226380 10.1111/j.1440-1746.2008.05762.x PMC3128252

[33] Yamaoka Y , Kikuchi S , El‐Zimaity HM , Gutierrez O , Osato MS , Graham DY . Importance of Helicobacter pylori oipA in clinical presentation, gastric inflammation, and mucosal interleukin 8 production. Gastroenterology. 2002;123:414‐424. 12145793 10.1053/gast.2002.34781

[34] Yamaoka Y , Kwon DH , Graham DY . A M(r) 34,000 proinflammatory outer membrane protein (oipA) of Helicobacter pylori. Proc Natl Acad Sci USA. 2000;97:7533‐7538. 10852959 10.1073/pnas.130079797 PMC16580

[35] Shakeri R , Malekzadeh R , Nasrollahzadeh D , et al. Multiplex H. pylori serology and risk of gastric cardia and noncardia adenocarcinomas. Cancer Res. 2015;75:4876‐4883. 26383162 10.1158/0008-5472.CAN-15-0556 PMC4792189

[36] Graham DY . Helicobacter pylori update: gastric cancer, reliable therapy, and possible benefits. Gastroenterology. 2015;148(719–731):e3. 10.1053/j.gastro.2015.01.040 PMC4375058 25655557

[37] Maeda M , Moro H , Ushijima T . Mechanisms for the induction of gastric cancer by Helicobacter pylori infection: aberrant DNA methylation pathway. Gastric Cancer. 2017;20:8‐15. 27718135 10.1007/s10120-016-0650-0

[38] Ajani JA , Lee J , Sano T , Janjigian YY , Fan D , Song S . Gastric adenocarcinoma. Nat Rev Dis Primers. 2017;3:17036. 28569272 10.1038/nrdp.2017.36

[39] Cover TL , Tummuru MK , Cao P , Thompson SA , Blaser MJ . Divergence of genetic sequences for the vacuolating cytotoxin among Helicobacter pylori strains. J Biol Chem. 1994;269:10566‐10573. 8144644

[40] Wroblewski LE , Peek RM Jr , Wilson KT . Helicobacter pylori and gastric cancer: factors that modulate disease risk. Clin Microbiol Rev. 2010;23:713‐739. 20930071 10.1128/CMR.00011-10 PMC2952980

[41] Jones KR , Jang S , Chang JY , et al. Polymorphisms in the intermediate region of VacA impact Helicobacter pylori‐induced disease development. J Clin Microbiol. 2011;49:101‐110. 21084502 10.1128/JCM.01782-10 PMC3020468

[42] Winter JA , Letley DP , Cook KW , et al. A role for the vacuolating cytotoxin, VacA, in colonization and Helicobacter pylori‐induced metaplasia in the stomach. J Infect Dis. 2014;210:954‐963. 24625807 10.1093/infdis/jiu154 PMC4136800

[43] Basso D , Zambon CF , Letley DP , et al. Clinical relevance of Helicobacter pylori cagA and vacA gene polymorphisms. Gastroenterology. 2008;135:91‐99. 18474244 10.1053/j.gastro.2008.03.041

[44] Chung C , Olivares A , Torres E , Yilmaz O , Cohen H , Perez‐Perez G . Diversity of VacA intermediate region among Helicobacter pylori strains from several regions of the world. J Clin Microbiol. 2010;48:690‐696. 20053862 10.1128/JCM.01815-09 PMC2832466

[45] Abdi E , Latifi‐Navid S , Latifi‐Navid H , Safarnejad B . Helicobacter pylori vacuolating cytotoxin genotypes and preneoplastic lesions or gastric cancer risk: a meta‐analysis. J Gastroenterol Hepatol. 2016;31:734‐744. 26648346 10.1111/jgh.13256

[46] Bakhti SZ , Latifi‐Navid S , Zahri S , Bakhti FS , Hajavi N , Yazdanbod A . Are Helicobacter pylori highly cytotoxic genotypes and cardia gastric adenocarcinoma linked? Lessons from Iran. Cancer Biomark. 2017;21:235‐246. 29036792 10.3233/CBM-170701 PMC13075733

[47] Bakhti SZ , Latifi‐Navid S , Zahri S . Unique constellations of five polymorphic sites of Helicobacter pylori vacA and cagA status associated with risk of gastric cancer. Infect Genet Evol. 2019;79:104167. 31891782 10.1016/j.meegid.2019.104167

[48] Wroblewski LE , Peek RM . Helicobacter pylori in gastric carcinogenesis: mechanisms. Gastroenterol Clin North Am. 2013;42:285‐298. 23639641 10.1016/j.gtc.2013.01.006 PMC3648881

[49] Higashi H , Yokoyama K , Fujii Y , et al. EPIYA motif is a membrane‐targeting signal of Helicobacter pylori virulence factor CagA in mammalian cells. J Biol Chem. 2005;280:23130‐23137. 15831497 10.1074/jbc.M503583200

[50] Noto JM , Peek RM . The Helicobacter pylori cag Pathogenicity Island. Methods Mol Biol. 2012;921:41‐50. 23015490 10.1007/978-1-62703-005-2_7 PMC3547679

[51] Salih BA , Bolek BK , Arikan S . DNA sequence analysis of cagA 3' motifs of Helicobacter pylori strains from patients with peptic ulcer diseases. J Med Microbiol. 2010;59:144‐148. 19850704 10.1099/jmm.0.014894-0

[52] Peek RM Jr , Miller GG , et al. Heightened inflammatory response and cytokine expression in vivo to cagA^+^ Helicobacter pylori strains. Lab Invest. 1995;73:760‐770. 8558837

[53] Peek RM Jr , Moss SF , et al. Helicobacter pylori cagA^+^ strains and dissociation of gastric epithelial cell proliferation from apoptosis. J Natl Cancer Inst. 1997;89:863‐868. 9196252 10.1093/jnci/89.12.863

[54] Chen CY , Wang FY , Wan HJ , et al. Amino acid polymorphisms flanking the EPIYA‐A motif of Helicobacter pylori CagA C‐terminal region is associated with gastric cancer in east China: experience from a single center. J Dig Dis. 2013;14:358‐365. 23517408 10.1111/1751-2980.12056

[55] Choi KD , Kim N , Lee DH , et al. Analysis of the 3' variable region of the cagA gene of Helicobacter pylori isolated in Koreans. Dig Dis Sci. 2007;52:960‐966. 17342405 10.1007/s10620-005-9030-z

[56] Jones KR , Joo YM , Jang S , et al. Polymorphism in the CagA EPIYA motif impacts development of gastric cancer. J Clin Microbiol. 2009;47:959‐968. 19158258 10.1128/JCM.02330-08 PMC2668329

[57] Kim YS , Kim N , Kim JM , et al. Helicobacter pylori genotyping findings from multiple cultured isolates and mucosal biopsy specimens: strain diversities of Helicobacter pylori isolates in individual hosts. Eur J Gastroenterol Hepatol. 2009;21:522‐528. 19373969 10.1097/meg.0b013e3283196af0

[58] Bastos J , Peleteiro B , Barros R , et al. Sociodemographic determinants of prevalence and incidence of Helicobacter pylori infection in Portuguese adults. Helicobacter. 2013;18:413‐422. 23725608 10.1111/hel.12061

[59] Yamaoka Y . Mechanisms of disease: Helicobacter pylori virulence factors. Nat Rev Gastroenterol Hepatol. 2010;7:629‐641. 20938460 10.1038/nrgastro.2010.154 PMC3137895

[60] Miftahussurur M , Yamaoka Y , Graham DY . Helicobacter pylori as an oncogenic pathogen, revisited. Expert Rev Mol Med. 2017;19:e4. 28322182 10.1017/erm.2017.4 PMC6905048

[61] Plummer M , van Doorn LJ , Franceschi S , et al. Helicobacter pylori cytotoxin‐associated genotype and gastric precancerous lesions. J Natl Cancer Inst. 2007;99:1328‐1334. 17728213 10.1093/jnci/djm120

[62] Epplein M , Butt J , Zhang Y , et al. Validation of a blood biomarker for identification of individuals at high risk for gastric cancer. Cancer Epidemiol Biomarkers Prev. 2018;27:1472‐1479. 30158280 10.1158/1055-9965.EPI-18-0582 PMC6279536

[63] Subsomwong P , Miftahussurur M , Vilaichone RK , et al. Helicobacter pylori virulence genes of minor ethnic groups in North Thailand. Gut Pathog. 2017;9:56. 29046726 10.1186/s13099-017-0205-x PMC5637267

[64] Acosta N , Quiroga A , Delgado P , Bravo MM , Jaramillo C . Helicobacter pylori CagA protein polymorphisms and their lack of association with pathogenesis. World J Gastroenterol. 2010;16:3936‐3943. 20712055 10.3748/wjg.v16.i31.3936 PMC2923768

[65] Higashi H , Tsutsumi R , Fujita A , et al. Biological activity of the Helicobacter pylori virulence factor CagA is determined by variation in the tyrosine phosphorylation sites. Proc Natl Acad Sci USA. 2002;99:14428‐14433. 12391297 10.1073/pnas.222375399 PMC137900

[66] Hatakeyama M . Oncogenic mechanisms of the Helicobacter pylori CagA protein. Nat Rev Cancer. 2004;4:688‐694. 15343275 10.1038/nrc1433

[67] Hatakeyama M , Higashi H . Helicobacter pylori CagA: a new paradigm for bacterial carcinogenesis. Cancer Sci. 2005;96:835‐843. 16367902 10.1111/j.1349-7006.2005.00130.x PMC11159386

[68] Honarmand‐Jahromy S , Siavoshi F , Malekzadeh R , Sattari TN , Latifi‐Navid S . Multiple repeats of Helicobacter pylori CagA EPIYA‐C phosphorylation sites predict risk of gastric ulcer in Iran. Microb Pathog. 2015;89:87‐92. 26408373 10.1016/j.micpath.2015.09.005

[69] Jang S , Jones KR , Olsen CH , et al. Epidemiological link between gastric disease and polymorphisms in VacA and CagA. J Clin Microbiol. 2010;48:559‐567. 19955279 10.1128/JCM.01501-09 PMC2815640

[70] Chomvarin C , Phusri K , Sawadpanich K , et al. Prevalence of cagA EPIYA motifs in Helicobacter pylori among dyspeptic patients in northeast Thailand. Southeast Asian J Trop Med Public Health. 2012;43:105‐115. 23082560

[71] Karlsson A , Ryberg A , Dehnoei MN , Borch K , Monstein HJ . Association between cagA and vacA genotypes and pathogenesis in a Helicobacter pylori infected population from South‐eastern Sweden. BMC Microbiol. 2012;12:129. 22747681 10.1186/1471-2180-12-129 PMC3520705

[72] Goh KL , Cheah PL , Md N , Quek KF , Parasakthi N . Ethnicity and H. pylori as risk factors for gastric cancer in Malaysia: A prospective case control study. Am J Gastroenterol. 2007;102:40‐45. 17100981 10.1111/j.1572-0241.2006.00885.x

[73] Ren S , Higashi H , Lu H , Azuma T , Hatakeyama M . Structural basis and functional consequence of Helicobacter pylori CagA multimerization in cells. J Biol Chem. 2006;281:32344‐32352. 16954210 10.1074/jbc.M606172200

[74] Sicinschi LA , Correa P , Peek RM , et al. CagA C‐terminal variations in Helicobacter pylori strains from Colombian patients with gastric precancerous lesions. Clin Microbiol Infec. 2010;16:369‐378. 19456839 10.1111/j.1469-0691.2009.02811.x PMC2837774

[75] Ahire D , Alston T , Raffaniello R . Variations in the multimerization region of the Helicobacter pylori cytotoxin CagA affect virulence. Oncol Lett. 2017;13:1444‐1450. 28454275 10.3892/ol.2017.5562 PMC5403285

[76] Ogorodnik E , Raffaniello RD . Analysis of the 3′‐variable region of the cagA gene from Helicobacter pylori strains infecting patients at New York City hospitals. Microb Pathog. 2013;56:29‐34. 23117095 10.1016/j.micpath.2012.10.003

[77] Latifi‐Navid S , Mohammadi S , Maleki P , et al. Helicobacter pylori vacA d1/‐i1 genotypes and geographic differentiation between high and low incidence areas of gastric cancer in Iran. Arch Iran Med. 2013;16:330‐337. 23725065

[78] Basiri Z , Safaralizadeh R , Bonyadi MJ , Somi MH , Mahdavi M , Latifi‐Navid S . Helicobacter pylori vacA d1 genotype predicts risk of gastric adenocarcinoma and peptic ulcers in Northwestern Iran. Asian Pac J Cancer Prev. 2014;15:1575‐1579. 24641370 10.7314/apjcp.2014.15.4.1575

[79] Mottaghi B , Safaralizadeh R , Bonyadi M , Latifi‐Navid S , Somi MH . Helicobacter pylori vacA i region polymorphism but not babA2 status associated to gastric cancer risk in northwestern Iran. Clin Exp Med. 2016;16(1):57–63. 25472424 10.1007/s10238-014-0327-0

[80] Shiota S , Matsunari O , Watada M , Yamaoka Y . Virulence factors or ancestral origin of Helicobacter pylori: which is a better predictor of gastric cancer risk? Gut. 2012;61:469‐470. 21610271 10.1136/gutjnl-2011-300317 PMC3808968

[81] Shiota S , Suzuki R , Matsuo Y , et al. Helicobacter pylori from gastric cancer and duodenal ulcer show same phylogeographic origin in the Andean region in Colombia. PLoS ONE. 2014;9:e105392. 25121764 10.1371/journal.pone.0105392 PMC4133377

[82] Matsunari O , Shiota S , Suzuki R , et al. Association between Helicobacter pylori virulence factors and gastroduodenal diseases in Okinawa, Japan. J Clin Microbiol. 2012;50:876‐883. 22189111 10.1128/JCM.05562-11 PMC3295155

[83] Kita M , Yokota K , Okada H , et al. The genetic diversity of Helicobacter pylori virulence genes is not associated with gastric atrophy progression. Acta Med Okayama. 2013;67:93‐98. 23603925 10.18926/AMO/49667

[84] Matsunari O , Miftahussurur M , Shiota S , et al. Rare Helicobacter pylori virulence genotypes in Bhutan. Sci Rep. 2016;6:22584. 26931643 10.1038/srep22584 PMC4773856

[85] Thorell K , Yahara K , Berthenet E , et al. Rapid evolution of distinct Helicobacter pylori subpopulations in the Americas. PLoS Genet. 2017;13:e1006546. 28231283 10.1371/journal.pgen.1006546 PMC5322909

[86] Persson C , Canedo P , Machado JC , El‐Omar EM , Forman D . Polymorphisms in inflammatory response genes and their association with gastric cancer: a HuGE systematic review and meta‐analyses. Am J Epidemiol. 2011;173:259‐270. 21178102 10.1093/aje/kwq370 PMC3105271

[87] Gianfagna F , de Feo E , van Duijn CM , Ricciardi G , Boccia S . A systematic review of meta‐analyses on gene polymorphisms and gastric cancer risk. Curr Genomics. 2008;9:361‐374. 19506726 10.2174/138920208785699544 PMC2691668

[88] Torres J , Correa P , Ferreccio C , et al. Gastric cancer incidence and mortality is associated with altitude in the mountainous regions of Pacific Latin America. Cancer Causes Control. 2013;24:249‐256. 23224271 10.1007/s10552-012-0114-8 PMC3697934

[89] Yamaoka Y , Graham DY . Helicobacter pylori virulence and cancer pathogenesis. Future Oncol. 2014;10:1487‐1500. 25052757 10.2217/fon.14.29 PMC4197059

[90] Barbosa HPM , Martins LC , dos Santos SEB , Demachki S , Assumpção M , de Oliveira Corvelo TC . Interleukin‐1 and TNF‐α polymorphisms and Helicobacter pylori in a Brazilian Amazon population. World Journal of Gastroenterology. 2009;15(12):1465–1471. 19322919 10.3748/wjg.15.1465 PMC2665140

[91] Ramis IB , Vianna JS , Gonçalves CV , von Groll A , Dellagostin OA , da Silva PEA . Polymorphisms of the IL‐6, IL‐8 and IL‐10 genes and the risk of gastric pathology in patients infected with Helicobacter pylori. Journal of Microbiology, Immunology and Infection. 2017;50(2):153–159. 10.1016/j.jmii.2015.03.002 25888319

[92] Sun X , Xu Y , Zhang F , Jing T , Han J , Zhang J . Association between the IL1B− 31C> T polymorphism and Helicobacter pylori infection in Asian and Latin American population: A meta‐analysis. Microbial pathogenesis. 2015;86:45–52. 26188264 10.1016/j.micpath.2015.07.010

[93] El‐Omar EM , Rabkin CS , Gammon MD , et al. Increased risk of noncardia gastric cancer associated with proinflammatory cytokine gene polymorphisms. Gastroenterology. 2003;124(5):1193–1201. 12730860 10.1016/s0016-5085(03)00157-4

[94] Puculek M , Machlowska J , Wierzbicki R , Baj J , Maciejewski R , Sitarz R . Helicobacter pylori associated factors in the development of gastric cancer with special reference to the early‐onset subtype. Oncotarget. 2018;9(57):31146–31162. 30123433 10.18632/oncotarget.25757 PMC6089554

[95] Hong J‐B , Zuo W , Wang A‐J , Lu N‐H . Helicobacter pylori Infection Synergistic with IL‐1β Gene Polymorphisms Potentially Contributes to the Carcinogenesis of Gastric Cancer. International journal of medical sciences. 2016;13(4):298–303. 27076787 10.7150/ijms.14239 PMC4829543

[96] Jakobsson M , Scholz SW , Scheet P , et al. Genotype, haplotype and copy‐number variation in worldwide human populations. Nature. 2008;451:998‐1003. 18288195 10.1038/nature06742

[97] Li JZ , Absher DM , Tang H , et al. Worldwide human relationships inferred from genome‐wide patterns of variation. Science. 2008;319:1100‐1104. 18292342 10.1126/science.1153717

[98] Popejoy AB , Fullerton SM . Genomics is failing on diversity. Nature. 2016;538:161‐164. 27734877 10.1038/538161a PMC5089703

[99] Gardy JL , Loman NJ . Towards a genomics‐informed, real‐time, global pathogen surveillance system. Nat Rev Genet. 2018;19:9‐20. 29129921 10.1038/nrg.2017.88 PMC7097748

[100] Goodwin S , McPherson JD , McCombie WR . Coming of age: ten years of next‐generation sequencing technologies. Nat Rev Genet. 2016;17:333‐351. 27184599 10.1038/nrg.2016.49 PMC10373632

[101] Conti DV , Wang K , Sheng X , et al. Two novel susceptibility loci for prostate cancer in men of African ancestry. J Natl Cancer Inst. 2017;109(8):djx084. 10.1093/jnci/djx084 PMC5448553 29117387

[102] Abdi E , Latifi‐Navid S , Zahri S , Yazdanbod A , Safaralizadeh R . Helicobacter pylori genotypes determine risk of non‐cardia gastric cancer and intestinal‐ or diffuse‐type GC in Ardabil: a very high‐risk area in Northwestern Iran. Microb Pathog. 2017;107:287‐292. 28390977 10.1016/j.micpath.2017.04.007

[103] Yakoob J , Abbas Z , Jafri W , Usman MW , Jafri F , Awan S . Comparison of the virulence markers of Helicobacter pylori and their associated diseases in patients from Pakistan and Afghanistan. Saudi J Gastroenterol. 2013;19:211‐218. 24045594 10.4103/1319-3767.118123 PMC3793472

[104] Khan A , Farooqui A , Raza Y , et al. Prevalence, diversity and disease association of Helicobacter pylori in dyspeptic patients from Pakistan. J Infect Dev Ctries. 2013;7:220‐228. 23493000 10.3855/jidc.2942

[105] Saribasak H , Salih BA , Yamaoka Y , Sander E . Analysis of Helicobacter pylori genotypes and correlation with clinical outcome in Turkey. J Clin Microbiol. 2004;42:1648‐1651. 15071020 10.1128/JCM.42.4.1648-1651.2004 PMC387623

[106] Hussein NR . Helicobacter pylori vacA d1 genotype is associated with gastric cancer but not peptic ulcers in Kurdistan region, Northern Iraq. Asian Pac J Cancer Prev. 2014;15:5965‐5966. 25081730 10.7314/apjcp.2014.15.14.5965

[107] El Khadir M , Alaoui Boukhris S , Benajah D‐A , et al. VacA and CagA status as biomarker of two opposite end outcomes of Helicobacter pylori Infection (gastric cancer and duodenal ulcer) in a moroccan population. PLoS ONE. 2017;12:e0170616. 28125638 10.1371/journal.pone.0170616 PMC5268467

[108] Memon AA , Hussein NR , Miendje Deyi VY , Burette A , Atherton JC . Vacuolating cytotoxin genotypes are strong markers of gastric cancer and duodenal ulcer‐associated Helicobacter pylori strains: a matched case‐control study. J Clin Microbiol. 2014;52:2984‐2989. 24920772 10.1128/JCM.00551-14 PMC4136171

[109] Miehlke S , Kirsch C , Agha‐Amiri K , et al. The Helicobacter pylori vacA s1, m1 genotype and cagA is associated with gastric carcinoma in Germany. Int J Cancer. 2000;87:322‐327. 10897035

[110] Ferreira RM , Machado JC , Letley D , et al. A novel method for genotyping the Helicobacter pylori vacA intermediate region directly in gastric biopsy specimens. J Clin Microbiol. 2012;50:3983‐3989. 23035185 10.1128/JCM.02087-12 PMC3502994

[111] Roman‐Roman A , Martinez‐Carrillo DN , Atrisco‐Morales J , et al. Helicobacter pylori vacA s1m1 genotype but not cagA or babA2 increase the risk of ulcer and gastric cancer in patients from Southern Mexico. Gut Pathog. 2017;9:18. 28413454 10.1186/s13099-017-0167-z PMC5390388

[112] Vinagre R , Vinagre IDF , Vilar ESA , Fecury AA , Martins LC . Helicobacter pylori infection and immune profile of patients with different gastroduodenal diseases. Arq Gastroenterol. 2018;55:122‐127. 30043859 10.1590/S0004-2803.201800000-21

[113] Stenkova AM , Lyalikova UV , Fayzullina NM , et al. Detection and genotyping of Helicobacter pylori gene vacA in children with gastroduodenal diseases and in adults with gastric cancer in Vladivostok. Bull Exp Biol Med. 2013;155:85‐88. 23667879 10.1007/s10517-013-2086-4

[114] Wei GC , Chen J , Liu AY , et al. Prevalence of Helicobacter pylori vacA, cagA and iceA genotypes and correlation with clinical outcome. Exp Ther Med. 2012;4:1039‐1044. 23226771 10.3892/etm.2012.704 PMC3494117

